# How do inpatients’ costs, length of stay, and quality of care vary across age groups after a new case-based payment reform in China? An interrupted time series analysis

**DOI:** 10.1186/s12913-023-09109-z

**Published:** 2023-02-15

**Authors:** Ya-jing Chen, Xin-yu Zhang, Xue Tang, Jia-qi Yan, Meng-cen Qian, Xiao-hua Ying

**Affiliations:** 1 School of Public Health, Fudan University, Shanghai, China; 2 Key Laboratory of Health Technology Assessment (Fudan University), Ministry of Health, Fudan University, 130 Dongan Road, Shanghai, China

**Keywords:** Payment reform, Case-based payment, Diagnosis-intervention packet, Cost, Out-of-pocket payment, Quality of care, Older adult

## Abstract

**Context:**

A patient classification-based payment system called diagnosis-intervention packet (DIP) was piloted in a large city in southeast China in 2018.

**Objective:**

This study evaluates the impact of DIP payment reform on total costs, out-of-pocket (OOP) payments, length of stay (LOS), and quality of care in hospitalised patients of different age.

**Methods:**

An interrupted time series model was employed to examine the monthly trend changes of outcome variables before and after the DIP reform in adult patients, who were stratified into a younger (18–64 years) and an older group (≥ 65 years), further stratified into young-old (65–79 years) and oldest-old (≥ 80 years) groups.

**Results:**

The adjusted monthly trend of costs per case significantly increased in the older adults (0.5%, *P* = 0.002) and oldest-old group (0.6%, *P* = 0.015). The adjusted monthly trend of average LOS decreased in the younger and young-old groups (monthly slope change: -0.058 days, *P* = 0.035; -0.025 days, *P* = 0.024, respectively), and increased in the oldest-old group (monthly slope change: 0.107 days, *P* = 0.030) significantly. The changes of adjusted monthly trends of in-hospital mortality rate were not significant in all age groups.

**Conclusion:**

Implementation of the DIP payment reform associated with increase in total costs per case in the older and oldest-old groups, and reduction in LOS in the younger and young-old groups without deteriorating quality of care.

## Introduction

In China, health care spending has been increasing rapidly since 2009, a growth rate of 14.1% from 2009 to 2019, higher than that of gross domestic product (GDP) [1], given the aging population [2]. By 2030, those older than 65 in China will go from 115 to 240 million, and those over 80 will soar from 12 million in 2000 to more than 40 million [3]. These demographic changes demand more healthcare, and the growth of per capita health care cost for older adults was increasing faster than other age groups [4]. Per capita health care cost for older adults was about 3–5 times higher than costs for other adults. It is estimated that China’s expenditure with older adults will almost double over the next three decades, increasing from 138 billion CNY in 2015 to 263 billion in 2050 [5].

In recent years, a new case-based payment system called diagnosis-intervention packet (DIP) was developed and piloted in China to increase transparency of resource consumption by standardising reimbursement and achieve greater efficiency by reducing unnecessary services. In 2018, DIP payment under regional global budget pilot reform launched in a large city in southeast China, relying on a new classification of patients based on combinations of the principal diagnosis, identified by the first four digits of ICD-10 code (International Classification of Diseases-10th revision) and procedures, identified by the ICD-9-CM3 code (International Classification of Diseases-Ninth Revision, Clinical Modification), resulting in a thorough classification of more than 10,000 groups. It differs from the previous fixed rate per admission with a cap on annual total compensation policy previously implemented in the city. It is a type of case-based payment under the regional global budget. Based on historical cost data, relative weights are assigned to patient groups to reflect market-wide (typically, at the prefecture-city level) resource utilization relative to different groups. Relative weights are then converted to payments according to the global insurance budget of the market [6]. Unlike the DRGs-based payment, DIP payment system mainly uses the ICD-10 and ICD-9-CM3 to identify the principal diagnosis and the procedure, respectively, while demographic and administrative variables (such as age, gender, and discharge status) are not used in classification. In addition, unlike the usual practice of the DRG-based payment in which the absolute reimbursements for each case are fixed in advance, the actual monetary payments are determined ex post based on the point value, although the relative values of DIP groups are fixed under the DIP-based payment [7].

Evidence from developed and developing countries showed that a case-based case-mix funding system might encourage inadequate practices, for example, selecting low-severity patients. Moreover, physicians might refuse complex patients because their treatment would lead to more resource consumption [8]. In China, the few studies that examined impacts of DRGs-based payment obtained mixed results on length of stay (LOS), mortality, cost and out-of-pocket (OOP) payments in all age groups [9–11]. In contrast, studies on this topic in European countries focused on quality of care and health care expenditure among inpatients. For instance, older patients, especially those aged 75 to 80, were reported to have longer LOS and higher total costs under DRGs-based payment, as well as more diagnoses per patient and a greater mortality than younger patients [12–14]. However, DesHarnais et al. [15] showed that age alone, in the absence of comorbidities or complications, only slightly increased hospital stay and resource consumption over most conditions for older Medicare patients. Importantly, international studies demonstrated that older groups present a challenge to the DRGs-based systems.

We hypothesized several changes following the DIP-based payment implementation. First, incentives for hospitals to decrease the probability of refusing elderly patients within the actual DIP, as the weight for older adults is recalibrated. Specifically, 1 percentage point will be added if the proportion of the number of inpatients of the elderly aged 60 or above in the designated medical institutions is equal to or greater than the average level of the pilot city. Second, expenditures per admission might increase, as the actual payments for cases treated are flexible rather than fixed in advance to contain total hospital reimbursements within the predetermined regional healthcare budget, thus the demand for medical services may be released. In addition, short anticipated length of stays, in order to cost savings. Finally, under the strong incentives of costs reducing, the quality might be affected.

The association between case-based payment, healthcare quality, healthcare expenditure and OOP payments in older adults have not been widely examined in China. Internationally, there is significant inaccessibility to service and unintended consequences with healthcare costs and quality of care among older patients under prospective payment systems [16–18]. If the case-based reimbursement scheme is inequitable, the current prospective payment system may provide a financial disincentive for hospitals and have adverse effects on hospital behaviour.

This study evaluated the impact of a DIP payment reform pilot in a large city in southeast China, in order to examine its association with health care outcomes (in-hospital mortality and LOS) along with its effect on total costs per case and OOP payments in different age groups, especially older patients. This study provides information to hospital administrators for achieving better health care services for older adults under the implementation of DIP-based global budget payment.

## Methods

### Data and population

We acquired de-identified patient-level discharge data of hospitalised patients in all contracted hospitals in the city from 2016 to 2019. The dataset included information on patient characteristics (e.g. age, sex, and insurance type), inpatient services (e.g. admission and discharge date, and discharge status), diagnoses, procedures and costs, and hospital level (tertiary, secondary and primary) and ownership (public and private).

The Health Security Administration of the city administers two main local social basic health insurance schemes, i.e. the urban employee basic medical insurance scheme (UEBMIS) and the residence basic medical insurance scheme (RBMIS), accounting for 7.52 million (50% of the population) and 4.96 million people (33%) in 2018, respectively [19]. Since the reimbursement rate of the RBMIS increased greatly in January 1^st^, 2018, coinciding with the time of the DIP reform and affecting OOP payments, we included only patients covered by the UEBMIS to identify net effects of the payment reform. To observe the impact of DIP reform across different ages of patients, we stratified them: younger (18–64 years) and an older group (≥ 65 years), further stratified into young-old (65–79 years) and oldest-old (≥ 80 years) group [20, 21].

### Study variables

We selected four outcome variables: 1) costs: total costs per case of discharged patients; 2) affordability of patients: OOP payments per case; 3) efficiency: average LOS; and 4) quality: in-hospital mortality rate. Total costs and out-of-pocket payments were adjusted to 2019 considering inflation using annual consumer price index of China [22]. LOS was calculated by the interval between admission and discharge dates. In-hospital mortality was identified by discharge status. Total costs, OOP payments and LOS were continuous variables and in-hospital mortality was constructed as a dichotomous variable (0 or 1) at the patient-level. We used covariates of patient characteristics: age, sex and Charlson Comorbidity Index (CCI), and hospital characteristics: accreditation level and ownership of hospitals. CCI was a measurement of patient severity based on comorbidities and was calculated according to ICD-10 codes of secondary diagnoses [23, 24].

### Statistical analysis

Patient characteristics and outcome variables were first compared before and after the DIP reform using *t*-test and chi-square test. Then we applied a quasi-experimental interrupted time series (ITS) study design to examine change of monthly trend of four outcome variables in hospitalised patients covered by the UEBMIS before (January 1^st^, 2016 to December 31^st^, 2017) and after (January 1^st^, 2018 to December 31^st^, 2019) the DIP reform. We used segmented regression model as specified:

1 $${Y}_{t}={\beta }_{0}+{\beta }_{1}{T}_{t}+{\beta }_{2}{DIP}_{t}+{\beta }_{3}{DIP}_{t}{T}_{t}+\alpha {X}_{t}+{\varepsilon }_{t}$$

where ${Y}_{t}$ represents the aggregated outcome variable in each month. Total costs per case and OOP payments per case were logarithmically transformed to adjust the skewed distribution. *T*_*t*_ is a monthly linear time trend of 48 months; *DIP*_*t*_ is a dummy variable, which equals 0 before the DIP reform and equals 1 after the reform; and *DIP*_*t*_*T*_*t*_ is their interaction. *X*_*t*_ is a vector of control variable at the year-month level, including the number of discharge cases, age, sex, CCI, hospital level, hospital ownership, and seasonality. The intercept ${\beta }_{0}$ represents the baseline level of the outcome variable, and ${\beta }_{1}$ is the monthly slope (trend) of the outcome variable before the DIP reform. ${\beta }_{2}$ and ${\beta }_{3}$ are change of the level and slope of the outcome variable after the reform, respectively. We handled the autocorrelation by fitting a Prais-Winsten estimation with the Durbin-Watson statistic, and used robust standard error [25, 26].

We first estimated the impacts on outcome variables in all adult patients covered by the UEBMIS. Then, we performed stratified analyses between the younger patients and the older ones, and between the young-old patients and oldest-old patients. We used 5% as the significance level. All analyses were conducted using Stata 16.0 for Windows.

## Results

### Descriptive statistics

Table 1 summarised the sample characteristics. We identified 1,721,889 discharge cases covered by the UEBMIS before the DIP reform, and 2,106,654 cases after the reform. The proportion of younger patients and oldest-old patients slightly increased after the reform. More than 80% and 90% of the patients were from the tertiary and public hospitals, respectively. In the whole sample, the total costs per case and OOP payments per case increased significantly (*P* = 0.000) after the DIP payment reform, while the average LOS and in-hospital mortality rate decreased significantly (*P* = 0.000). In different age groups, the change patterns of outcome variables were similar, except for total costs per case and OOP payment per case in patients aged 80 and above, which decreased after the reform; and average LOS in this group increased after the DIP reform. When comparing across age groups, total cost per case was higher in older adults, while OOP payments per case was the lowest in the oldest-old group. Average LOS and in-hospital mortality rate both increased with age.

**Table 1:** Sample characteristics of hospitalized patients covered by the urban employee basic medical insurance, 2016–2019

Variables	Before DIP reform, 2016–2017	After DIP reform, 2018–2019	*P* value
**Patient characteristics**			
Discharge cases, No	1,721,889	2,106,654	
Age, No. (%)			0.000
18–64	1,032,761 (59.98)	1,276,735 (60.60)	
65–79	444,244 (25.80)	520,388 (24.71)	
≥ 80	244,884 (14.22)	309,531 (14.69)	
Male sex, No. (%)	783,263 (45.49)	972,573 (46.17)	0.000
Charlson Comorbidity Index, mean (SD)	0.77 (1.33)	0.96 (1.52)	0.000
Hospital level, No. (%)			0.000
Tertiary	1,431,706 (83.15)	1,749,473 (83.05)	
Secondary	233,494 (13.56)	278,935 (13.24)	
Primary	56,689 (3.29)	78,246 (3.71)	
Hospital ownership, No. (%)			0.000
Public	1,631,031 (94.72)	1,990,426 (94.48)	
Private	90,858 (5.28)	116,228 (5.52)	
**Patient outcomes, mean (SD)**			
All patients			
Total costs per case (RMB)	16,187.40 (19,901.94)	16,657.84 (19,705.70)	0.000
Out-of-pocket payments per case (RMB)	6373.27 (12,672.19)	6524.58 (12,427.80)	0.000
Length of stay (days)	9.68 (11.49)	9.32 (11.27)	0.000
In-hospital mortality (%)	1.39 (11.71)	1.28 (11.25)	0.000
Age group: younger (18–64)			
Total costs per case (RMB)	15,521.60 (19,404.23)	16,108.73 (19,430.28)	0.000
Out-of-pocket payments per case (RMB)	6528.32 (12,518.91)	6873.86 (12,360.19)	0.000
Length of stay (days)	8.49 (10.07)	7.96 (9.81)	0.000
In-hospital mortality (%)	0.64 (7.96)	0.54 (7.34)	0.000
Age group: young-old (65–79)			
Total costs per case (RMB)	17,885.85 (21,436.15)	18,538.62 (21,498.90)	0.000
Out-of-pocket payments per case (RMB)	6813.50 (13,717.18)	6988.48 (13,618.09)	0.000
Length of stay (days)	10.63 (11.80)	10.26 (11.19)	0.000
In-hospital mortality (%)	1.65 (12.74)	1.51 (12.19)	0.000
Age group: oldest-old (80 +)			
Total costs per case (RMB)	15,921.15 (1884.18)	15,770.71 (17,346.11)	0.002
Out-of-pocket payments per case (RMB)	4926.84 (11,150.59)	4301.29 (10,166.95)	0.000
Length of stay (days)	12.97 (15.16)	13.36 (15.21)	0.000
In-hospital mortality (%)	4.10 (19.83)	3.96 (19.51)	0.010

DIP denoted the Diagnosis-Intervention Packet payment reform. Total costs and out-of-pocket payments were adjusted to 2019 considering inflation using annual consumer price index of China

### Total costs per case

**Fig. 1:**
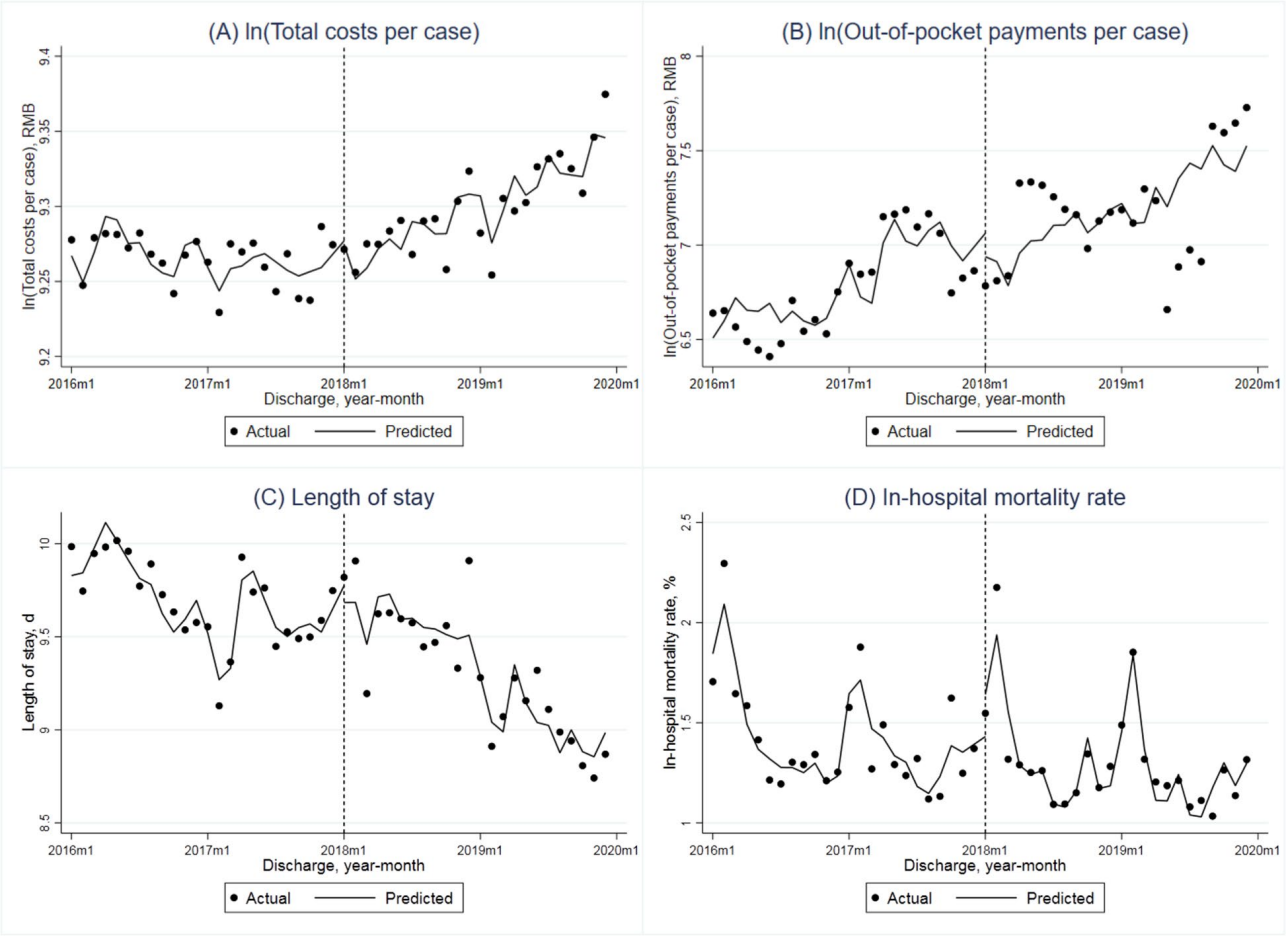
Monthly trends in adjusted total costs per case (in log form), out-of-pocket payments per case (in log form), length of stay and in-hospital mortality rate of hospitalized adult patients covered by the urban employee basic medical insurance

**Fig. 2:**
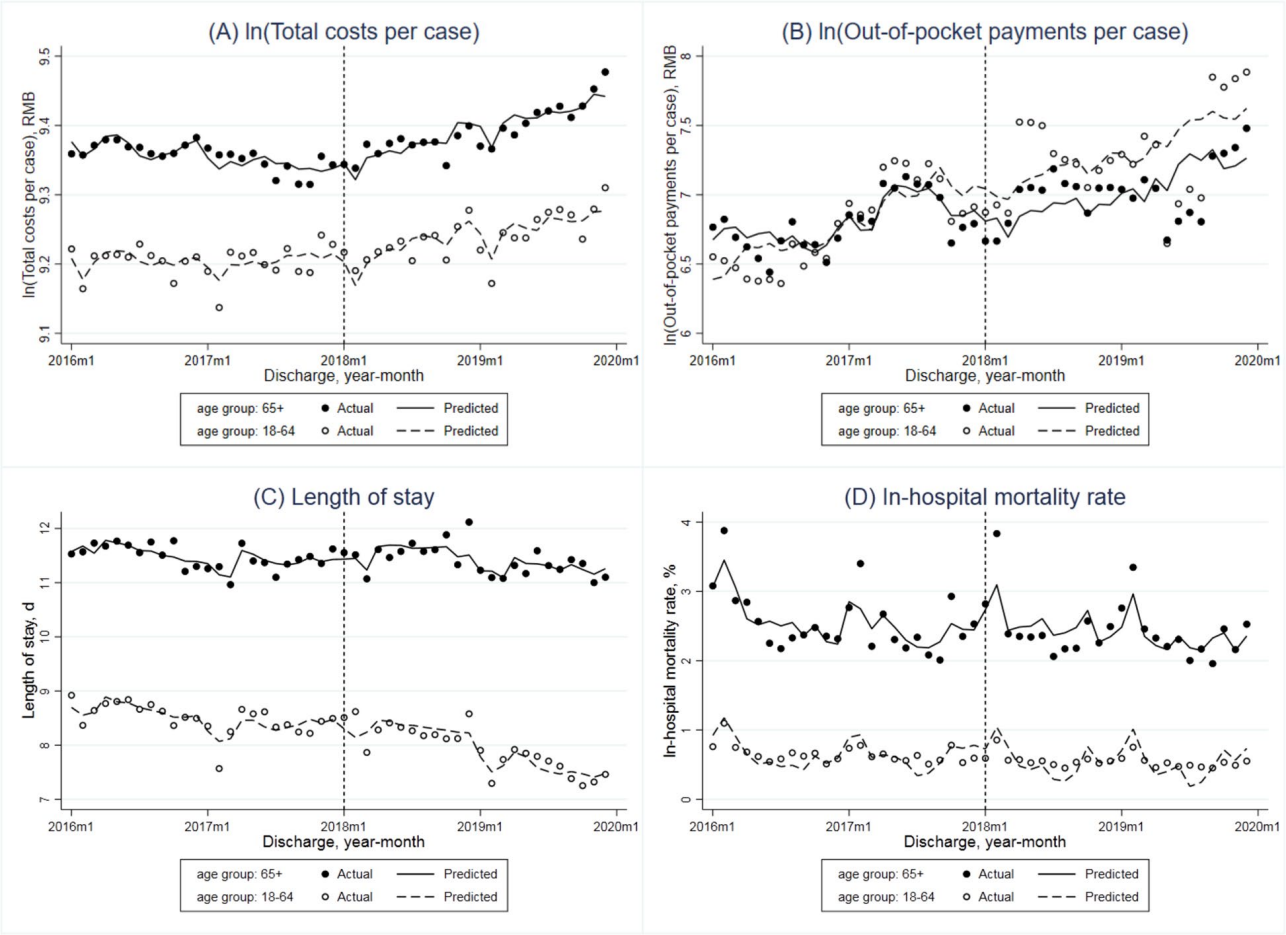
Monthly trends in adjusted total costs per case (in log form), out-of-pocket payments per case (in log form), length of stay and in-hospital mortality rate in hospitalized patients covered by the urban employee basic medical insurance with different age groups (younger patients aged 18–64 vs older patients aged 65 +)

**Fig. 3:**
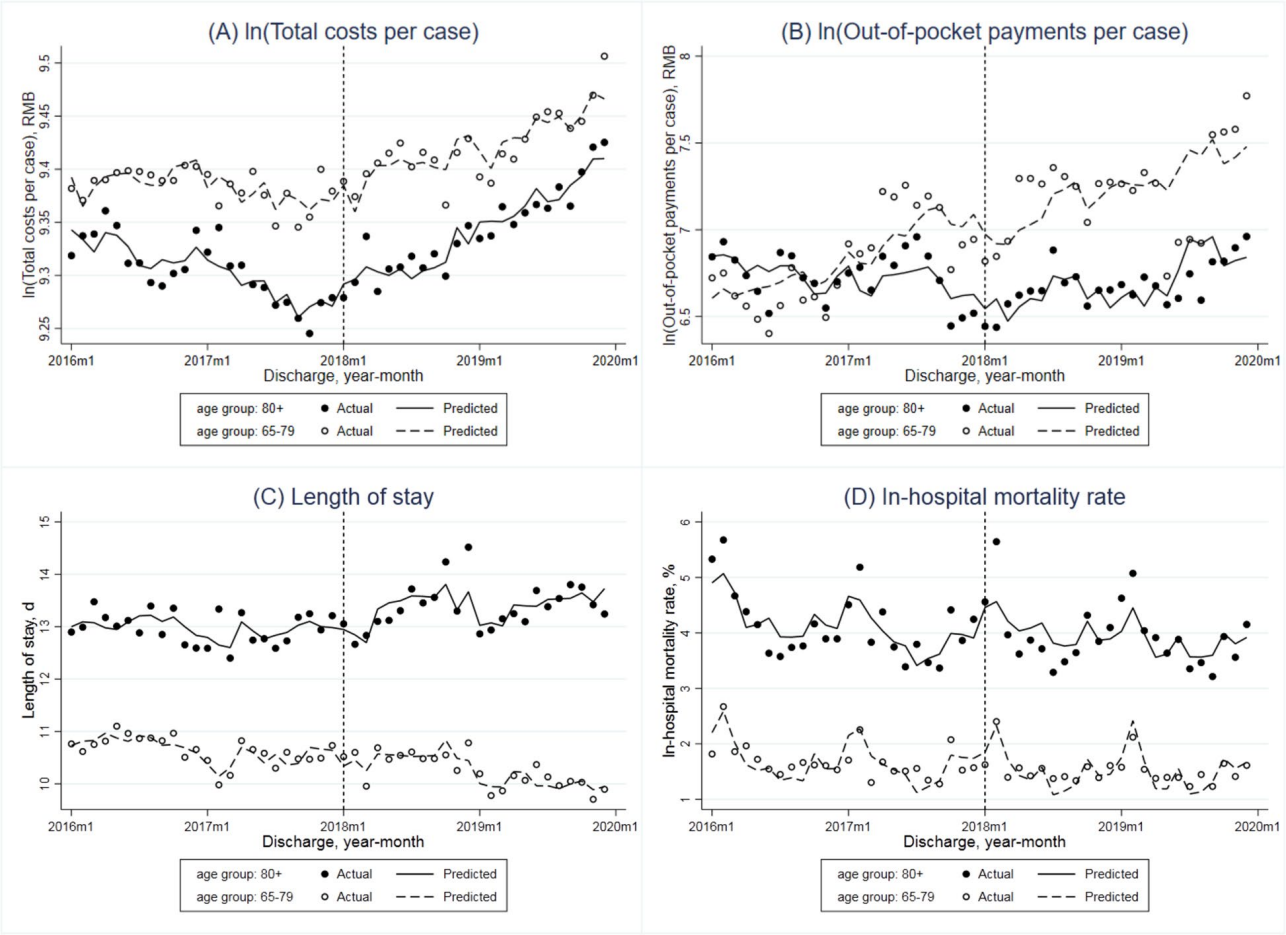
Monthly trends in adjusted total costs per case (in log form), out-of-pocket payments per case (in log form), length of stay and in-hospital mortality rate in hospitalized patients covered by the urban employee basic medical insurance aged above 65 years (young-old patients aged 65–79 vs oldest-old patients aged 80 +)

The adjusted monthly trend of total costs per case in all adult patients covered by the UEBMIS showed an insignificant reduction (-0.3% per month, *P* = 0.067) in the pre-DIP period (Table 2 and Fig. 1A). The DIP reform resulted in a significant increase of its immediate level at 4.1% (*P* = 0.016) and increase of monthly trend at 0.5% per month (*P* = 0.060). In the stratified analysis (Fig. 2A), the level of costs per case was always higher in older adults compared to the younger group. Before the reform, the adjusted monthly trend was not significant in any group. While after the reform, total costs per case in older adults showed a significant increase of the immediate level (2.8%, *P* = 0.013) and the monthly slope (0.5%, *P* = 0.002). When comparing the young-old and oldest-old groups (Fig. 3A), total costs per case of the latter were lower than the former. The monthly trend both increased in the post-DIP period, but only increased significantly in the oldest-old patients (0.6%, *P* = 0.015).

**Table 2:** Interrupted time series (ITS) analyses for total costs per case, out-of-pocket payments per case, length of stay and in-hospital mortality rate of hospitalized patients covered by the urban employee basic medical insurance before and after the DIP reform

Indicators	Baseline monthly slope (β1)		Step change (β2)		Monthly slope change (β3)		Constant (β0)	
	Estimate (95%CI)	*P* value	Estimate (95%CI)	*P* value	Estimate (95%CI)	*P* value	Estimate (95%CI)	*P* value
**All patients**								
ln (Total costs per case)	-0.003 (-0.006, 0.000)	0.067	0.041 (0.008, 0.074)	0.016	0.005 (0.000, 0.010)	0.060	7.820 (6.908, 8.732)	0.000
ln (Out-of-pocket payments per case)	0.016 (-0.015, 0.047)	0.301	-0.298 (-0.661, 0.065)	0.105	-0.011 (-0.074, 0.051)	0.713	-2.228 (-20.673, 16.217)	0.808
Length of stay	-0.051 (-0.079, -0.023)	0.001	0.271 (-0.019, 0.561)	0.066	-0.008 (-0.051, 0.035)	0.709	-3.287 (-12.478, 5.903)	0.472
In-hospital mortality rate	-0.015 (-0.033, 0.003)	0.093	-0.084 (-0.278, 0.110)	0.385	0.005 (-0.026, 0.036)	0.739	-2.359 (-7.588, 2.871)	0.366
**Age groups: younger vs older**								
Age group: younger (18–64)								
ln (Total costs per case)	0.001 (-0.003, 0.004)	0.678	0.029 (-0.005, 0.063)	0.092	-0.001 (-0.006, 0.003)	0.584	7.794 (6.695, 8.894)	0.000
ln (Out-of-pocket payments per case)	0.036 (0.006, 0.067)	0.022	-0.325 (-0.731, 0.081)	0.114	-0.014 (-0.094, 0.066)	0.723	-7.160 (-23.097, 8.776)	0.368
Length of stay	-0.019 (-0.055, 0.017)	0.286	0.351 (-0.005, 0.706)	0.053	-0.058 (-0.111, -0.004)	0.035	-2.603 (-11.348, 6.142)	0.550
In-hospital mortality rate	-0.011 (-0.020, -0.002)	0.021	-0.108 (-0.203, -0.013)	0.027	0.012 (-0.000, 0.024)	0.058	1.075 (-1.372, 3.522)	0.378
Age group: older (65 +)								
ln (Total costs per case)	-0.001 (-0.003, 0.001)	0.255	0.028 (0.006, 0.049)	0.013	0.005 (0.002, 0.008)	0.002	10.273 (9.845, 10.701)	0.000
ln (Out-of-pocket payments per case)	0.004 (-0.021, 0.029)	0.734	-0.163 (-0.423, 0.097)	0.211	-0.008 (-0.058, 0.041)	0.738	2.227 (-6.863, 11.317)	0.622
Length of stay	-0.059 (-0.081, -0.038)	0.000	0.360 (0.101, 0.619)	0.008	0.022 (-0.013, 0.057)	0.205	10.346 (3.064, 17.627)	0.007
In-hospital mortality rate	-0.013 (-0.038, 0.012)	0.303	-0.240 (-0.481, 0.001)	0.051	-0.030 (-0.080, 0.020)	0.229	-8.115 (-16.201, -0.028)	0.049
**Age groups: young-old vs oldest-old**								
Age group: young-old (65–79)								
ln (Total costs per case)	0.000 (-0.002, 0.003)	0.923	0.026 (-0.007, 0.060)	0.122	0.003 (-0.001, 0.006)	0.106	10.158 (9.511, 10.804)	0.000
ln (Out-of-pocket payments per case)	0.011 (-0.016, 0.038)	0.400	-0.134 (-0.422, 0.154)	0.351	-0.031 (-0.085, 0.024)	0.259	0.394 (-7.830, 8.617)	0.923
Length of stay	-0.038 (-0.053, -0.022)	0.000	0.249 (-0.017, 0.515)	0.066	-0.025 (-0.047, -0.004)	0.024	7.559 (0.997, 14.122)	0.025
In-hospital mortality rate	-0.010 (-0.026, 0.005)	0.184	-0.144 (-0.321, 0.033)	0.108	-0.014 (-0.040, 0.012)	0.274	-4.721 (-9.840, 0.397)	0.070
Age group: oldest-old (80 +)								
ln (Total costs per case)	-0.001 (-0.004, 0.002)	0.524	0.028 (-0.008, 0.063)	0.127	0.006 (0.001, 0.010)	0.015	9.398 (8.598, 10.198)	0.000
ln (Out-of-pocket payments per case)	-0.010 (-0.028, 0.008)	0.255	-0.012 (-0.264, 0.240)	0.923	0.025 (-0.001, 0.051)	0.057	5.781 (0.401, 11.161)	0.036
Length of stay	-0.097 (-0.152, -0.041)	0.001	0.622 (-0.002, 1.246)	0.051	0.107 (0.011, 0.204)	0.030	7.972 (-5.376, 21.320)	0.233
In-hospital mortality rate	-0.039 (-0.096, 0.019)	0.182	-0.233 (-0.836, 0.370)	0.437	-0.009 (-0.115, 0.098)	0.869	-5.736 (-22.689, 11.216)	0.497

DIP denoted the Diagnosis-Intervention Packet payment reform; CI the confidence interval. Total costs and out-of-pocket payments were adjusted to 2019 considering inflation using annual consumer price index of China. Total costs per case and out-of-pocket payments per case were logarithmically transformed in the ITS model. ITS analyses controlled for number of discharge cases, age, sex, Charlson Comorbidity Index of patients, hospital level, hospital ownership and seasonality, with robust standard errors

### Out-of-pocket payments per case

OOP payments per case in all patients showed an increase while not significant monthly trend (1.6%, *P* = 0.301) before the DIP payment reform, and the change of its monthly slope was negative but not significant (-1.1%, *P* = 0.713) either (Table 2 and Fig. 1B). The baseline monthly trends and monthly slope changes in the younger, older adult and young-old groups showed a similar pattern with the whole sample (Figs. 2B and 3B). While in the oldest-old group, OOP payments per case decreased by 1.0% per month (*P* = 0.255) before the reform and increased by 2.5% per month (*P* = 0.057) after the DIP payment reform occurred.

### Average length of stay

The average LOS significantly decreased 0.051 days per month before the DIP payment reform (*P* = 0.001), and this declining trend remained unchanged after the reform (-0.008 days per month, *P* = 0.709) overall (Table 2 and Fig. 1C). The adjusted average LOS both decreased for younger and older groups but was only significant for older adults (0.019 days, *P* = 0.286; 0.059 days, *P* = 0.000, respectively) before the reform. After the reform, the average LOS only decreased significantly in the younger group (0.058 days, *P* = 0.035) (Fig. 2C). The average LOS in the young-old group showed a significantly declining baseline monthly trend (0.038 days, *P* = 0.000) and a significantly decreasing monthly trend change (0.025 days, *P* = 0.024) associated with the DIP reform. In the oldest-old group, the average LOS also decreased significantly in the baseline period (0.097 days per month, *P* = 0.001), while showed a significant positive change of monthly slope (0.107 days per month, *P* = 0.030) in the post-reform period (Fig. 3C).

### In-hospital mortality rate

The overall adjusted in-hospital mortality rate presented a descending, but not significant, monthly trend before the DIP payment reform (Table 2 and Fig. 1D). After the reform, the immediate level change and monthly slope change were both not significant. In each age group, the in-hospital mortality rate showed a similar decreasing trend in the baseline, monthly slope (younger group: -0.011 percentage points, *P* = 0.021; elderly group: -0.013 percentage points, *P* = 0.303; young-old group: -0.010 percentage points, *P* = 0.184; oldest-old group: -0.039 percentage points, *P* = 0.182), and the monthly slope change after the DIP payment reform was not significant (younger group: 0.012 percentage points, *P* = 0.058; elderly group: -0.030 percentage points, *P* = 0.229; young-old group: -0.014 percentage points, *P* = 0.274; oldest-old group: -0.009 percentage points, *P* = 0.869) (Figs. 2D and 3D).

## Discussion

In this study, the implementation of DIP-based payment system was associated with significantly increase of monthly trend of total costs per case in the whole sample and older adult group. OOP payments presented an upward trend in the oldest-old group after the DIP-based payment. DIP reform resulted in a slight reduction of monthly trend of LOS in the young-old group and a significant increase in the oldest-old group. The in-hospital mortality showed a monthly downward trend in the older group after the DIP-based payment, while the result was not statistically significant.

The supply of medical services among the older group might increase in the period after the reform, thus improving patient accessibility. Total costs per case tend to be greatest among oldest-old group—associated with long lengths of stay and more intensive medical investments. The incidence of comorbidities and functional impairment is higher in older adults: oldest-old patients had more diagnoses and higher severities and the cost typically exceed those in other age groups [27]. Previous studies arrived at similar conclusions, suggesting that resource use within DRGs-based payment may vary according to health severity [28–31]. Current findings support the view that DIP-based payment system will account for the most differences in costs.

DIP-based global payment system implementation was associated with an increase of monthly trend in OOP payment among the oldest-old group, but no significant differences were found, possibly since DIP payment reform itself has no inherent incentive for changes in OOP payment. It also implies that physicians did not significantly increase OOP use, not placing the burden on patients, despite the increase in the annual total costs per case. The DIP payment reform implementation might have the smaller effect of relieving the financial burden on severe patients. Furthermore, although there is a risk of hospitals mainly focus on patients whose treatment costs are borne by themselves, and reject or just avoid Medicare patients, our study did not observe this phenomenon.

Changes in LOS were observed in young-old groups. The implementation of DIP-based payment may have stimulated behavioural changes leading to efficient discharge planning, higher reimbursement for older adults with higher severity [26]. On one hand, the increased severity of diseases found in young-old patient group may be mainly chronic and lead to more frequent discharges rather than more intensive care during individual discharges. On the other hand, compared with increasing LOS, more cost-effective mechanisms might be provided by the hospital for young-old patients to reduce mortality. Further, hospitals have the choice of either providing more nursing care (or other support) for patients over 80 or keep them longer under DIP.

Several factors may be behind the monthly downward trend of in-hospital mortality in the older Medicare patient group, partly because a shorter LOS per se is due to less time facing risks. Hospitals may tend to develop clinical pathways for more efficient care delivery without deteriorating the healthcare outcomes under the implication of DIP [32]. However, the previous literature does not examine the impact of DRG-based payment systems on in-hospital mortality across different ages. Quality outcomes, in terms of mortality, are helpful indicators to evaluate DRGs-based payment but have been criticised as insufficiently sensitive for healthcare quality [33].

The DIP reform has received the attention of policymakers in China. Current findings suggest that DIP-based payment can achieve real changes in the delivery of health care in hospitals. There is no discrimination against specific types of patients. Our results suggest that hospital reimbursement would be more equitable if age (especially ≥ 65 years) was considered in determining hospital reimbursement. Importantly, DIP-based payment facilitates the comparison of the difference in treatment costs between different medical institutions for the same illness combination, effectively promoting the professional division of labour and competition among medical institutions in the region, which can contribute towards assessing and supervising of competent departments [34]. This is the first study to evaluate the effect of DIP-based payment on cost, quality of care, and OOP payments across different age groups. It can enable the development of efficient and appropriate healthcare strategies to improve care quality. Moreover, it encourages further large-scale, multicentre studies.

Our study has some limitations. First, the sample are patients covered by the urban employee basic health insurance scheme (UEBHIS), limiting the generalisability. But individuals with UEBHIS represent most of the medical insurance population and demonstrated the greatest effect of DIP care quality and cost. Second, our study included only a short-term implementation period of the DIP-based global payment. Future studies are needed to evaluate the long-term impact of the DIP-based global system on cost, quality, and OOP payments. Third, our study included patients at all age groups but did not consider possible variations in health during different ages. Fourth, findings are likely to be influenced by additional factors, health status, severity of illness and social settings, which should be explored.

## Conclusion

The DIP-based payment system slightly increased the total cost per case of older and oldest-old adults, and reduced LOS of younger and young-old patients without deteriorating quality of life. The oldest-old group had more diagnoses and higher admission severity of diagnosis per patient, demonstrating higher OOP payment and longer LOS. These findings suggested that the DIP-based payment system may be adequate for older adults and support the continued implementation and enlargement of the DIP-based payment system in China, given its potential for inducing a shift in hospital supply services. Further studies are required to evaluate the association of the DIP-based payment system with in-hospital care to improve both the effectiveness and medical quality of the health care system.

## Data Availability

The data that support the findings of this study are available from Guangzhou Healthcare Security Administration but restrictions apply to the availability of these data, which were used under license for the current study, and so are not publicly available. Data are however available from the corresponding author upon reasonable request and with permission of Guangzhou Healthcare Security Administration.

## Abbreviations

DIP: Diagnosis-intervention packet
OOP: Out-of-pocket payments
LOS: Length of Stay
GDP: Gross Domestic Product
ICD-10 code: International Classification of Diseases-10th revision
ICD-9-CM3 code: International Classification of Diseases, Ninth Revision, Clinical Modification
UEBMIS: The Urban Employee Basic Medical Insurance Scheme
RBMIS: The Residence Basic Medical Insurance Scheme
CCI: Charlson Comorbidity Index
ITS: Interrupted Time Series

## References

[1] National Health Commission of the People's Republic of China. China Health Statistics Yearbook 2020. http://www.nhfpcgovcn/zwgkzt/tjnj/listshtml. Accessed 23 Sept 2020.

[2] Getzen TE (1992). Population Aging and the Growth of Health Expenditures. J Gerontol.

[3] Population Division of Department of Economic and Social Affairs of United Nations Secretariat. World Population Outlook: 2008 Revision. http://esaunorg/unpp.

[4] Wang C, Li F, Wang L, Zhou W, Jin C (2017). The impact of population aging on medical expenses: A big data study based on the life table. Biosci Trends.

[5] Hyjm A, Xl B, Qlxcd E, Shu C, Wu MC (2020). The association between frailty and healthcare expenditure among Chinese older adults. J Am Med Dir Assoc.

[6] Qian M, Zhang X, Chen Y, Xu S, Ying X (2021). The pilot of a new patient classification-based payment system in China: The impact on costs, length of stay and quality. Social Science Medicine.

[7] Yi L, Hq F, Ling L, Wy C (2021). Hospital response to a case-based payment scheme under regional global budget: The case of Guangzhou in China. Soc Sci Med.

[8] Yip WC, Meng Q, Chen W, Sun X (2010). Realignment of incentives for health-care providers in China. Lancet.

[9] Hu W, Yeh C, Shiao A, Tu T (2015). Effects of diagnosis-related group payment on health-care provider behaviors: a consecutive three-period study. J Chin Med Assoc.

[10] Jian W, Lu M, Liu G, Chan KY, Poon AN (2019). Beijing's diagnosis-related group payment reform pilot: Impact on quality of acute myocardial infarction care. Soc Sci Med.

[11] Jian W, Lu M, Chan KY (2015). Payment reform pilot in Beijing hospitals reduced expenditures and out-of-pocket payments per admission. Health Aff.

[12] Jencks SF, Kay T (1987). Do frail, disabled, poor, and very old Medicare beneficiaries have higher hospital charges?. JAMA.

[13] Muñoz E, Rosner F, Chalfin D, Goldstein J, Margolis IB, Wise L (1988). Financial risk and hospital cost for elderly patients. Age- and non-age-stratified medical diagnosis related groups. Arch of Intern Med.

[14] Castiel D, Bréchat P (2009). Social deprivation and public hospital: for a social DRG. Presse Medicale.

[15] Desharnais S, Chesney J, Fleming S (1988). Should DRG assignment be based on age?. J Med Care.

[16] Leu A, Wepf H, Elger B, Wangmo T (2018). Experts’ perspectives on SwissDRG: second class care for vulnerable patient groups?. Health Policy.

[17] Goldberg SC, Estes CL (1990). Medicare DRGs and post-hospital care for the elderly: does out of the hospital mean out of luck?. J Appl Gerontol.

[18] Lim SC, Doshi V, Castasus B, Lim JKH, Mamun K (2006). Factors causing delay in discharge of elderly patients in an acute care hospital. Ann Acad Med Singap.

[19] Guangzhou Municipal Statistics Bureau, Guangzhou Survey Office of National Bureau of Statistics. Guangzhou Statistical Yearbook (2019): Beijing: China Statistics Press 2019.

[20] Zhao Y, Xu X, Dupre ME, Xie Q, Gu D (2020). Individual-level factors attributable to urban-rural disparity in mortality among older adults in China. BMC Public Health.

[21] Zeng Y, Feng Q, Hesketh T (2017). Survival, disabilities in activities of daily living, and physical and cognitive functioning among the oldest-old in China: a cohort study. Lancet.

[22] National Bureau of Statistics of China (2022). China Statistical Yearbook (2022).

[23] Charlson ME, Pompei P, Ales KL, Mackenzie CR (1987). A new method of classifying prognostic comorbidity in longitudinal studies: development and validation. J Chronic Dis.

[24] Sundararajan V, Henderson T, Perry C, Muggivan A, Quan H, Ghali WA (2004). New ICD-10 version of the Charlson comorbidity index predicted in-hospital mortality. J Clin Epidemiol.

[25] Linden A (2015). Conducting interrupted time-series analysis for single- and multiple-group comparisons. Stata Journal.

[26] Kutz A, Gut L, Ebrahimi F, Wagner U, Schuetz P, Mueller B (2019). Association of the Swiss Diagnosis-Related Group Reimbursement System With Length of Stay, Mortality, and Readmission Rates in Hospitalized Adult Patients. JAMA Netw Open.

[27] Muoz E, Rosner F, Chalfin D, Goldstein J, Wise L (1989). Age, Resource Consumption, and Outcome for Medical Patients at an Academic Medical Center. Arch Intern Med.

[28] Rosenthal GE, Landefeld CS (1993). Do older Medicare patients cost hospitals more? Evidence from an academic medical center. Arch Intern Med.

[29] Horn SD, Bulkley G, Sharkey PD, Chambers AF, Horn RA, Schramm C (1985). Interhospital differences in severity of illness. Problems for prospective payment based on diagnosis-related groups (DRGs). N Engl J Med.

[30] Fulop M (1987). The Frail, the Old, and Diagnosis Related Groups. J Am Med Assoc.

[31] Horn SD, Horn RA, Sharkey PD (1984). The Severity of Illness Index as a severity adjustment to diagnosis-related groups. Health Care Financ Rev.

[32] Odderson IR, McKenna BS (1993). A model for management of patients with stroke during the acute phase. Outcome and economic implications. Stroke.

[33] Fischer C, Lingsma HF, Mheen M, Kringos DS, Steyerberg EW (2014). Is the Readmission Rate a Valid Quality Indicator? A Review of the Evidence. PLoS ONE.

[34] Ying Y (2021). DIP & DRG: similarities and differences. China Health Insurance China Health Insurance.

